# Intraventricular endoscopic surgery

**DOI:** 10.3171/2023.1.FOCVID2311

**Published:** 2023-04-01

**Authors:** Mark G. Hamilton, Ahmed K. Toma, Charles Teo, Caroline Hayhurst, Mark Souweidane

**Affiliations:** 1 Department of Clinical Neurosciences, Division of Neurosurgery, University of Calgary Cumming School of Medicine, Calgary, Alberta, Canada;; 2 Victor Horsley Department of Neurosurgery, National Hospital for Neurology & Neurosurgery, Queen Square, London, United Kingdom;; 3 Centre for Minimally Invasive Neurosurgery, Sydney, Australia;; 4 Department of Neurosurgery, University Hospital of Wales, Cardiff, Wales, United Kingdom; and; 5 Weill Cornell Medicine Brain & Spine Center, New York, New York

hydrocephalus in adults comprises a diverse grouping of etiologies, pathophysiology, diagnostic criteria, and treatment needs involving the CSF pathways. Further, hydrocephalus is more common in adults than in children,^1^ and surgical procedures for hydrocephalus in adults in the US are annually performed almost five times more frequently than in pediatric patients.^2^ A previously described pragmatic, clinically oriented organizational scheme helps to ensure we distinguish four types of patients within the spectrum of adult hydrocephalus: 1) transitional patients (previously diagnosed with hydrocephalus and treated as children), 2) patients with previously unrecognized congenital hydrocephalus, 3) patients with acquired hydrocephalus with an identifiable etiology (e.g., subarachnoid hemorrhage, cerebral trauma, or infection), and 4) patients with suspected or proven idiopathic normal pressure hydrocephalus.^3^ There are distinct differences in the diagnosis, treatment, and outcomes of these four patient groups.

This issue of *Neurosurgical Focus: Video* provides a series of excellent videos that each deal with a part of the spectrum of disorders and lesions affecting the CSF pathways. The most common neuroendoscopic procedure, endoscopic third ventriculostomy, while frequently undertaken as a sole procedure, is not addressed as a specific topic. However, there are excellent, compelling presentations that depict the use of image guidance to assist in the endoscopic management of complex hydrocephalus and a technique to expand endoscopic access to the third ventricle with the opening of the choroidal fissure. Other videos describe the fenestration of a giant arachnoid cyst, the resection of a colloid cyst, and the resection of tumors of the third and lateral ventricles. These videos will illustrate some of the capabilities of intraventricular neuroendoscopy, and we hope they will inspire and teach.

This is the first time *Neurosurgical Focus: Video* is exclusively devoted to intraventricular endoscopic surgery. It is noteworthy that the April issue of *Neurosurgical Focus* deals with adult hydrocephalus. This is especially relevant given that most intraventricular endoscopic surgery is usually undertaken against the backdrop of hydrocephalus. The synchronization of the April 2023 issues of* Neurosurgical Focus *and* Neurosurgical Focus: Video* was done to recognize the advancements that have occurred in the care of patients with hydrocephalus and hopefully provide an opportunity to appreciate diagnostic and management issues from different perspectives.

## Disclosures

Dr. Hamilton reported personal fees from Integra Canada and grants from Alberta Innovates "Reducing Shunt and CSF-Drain Related Infections" outside the submitted work.
